# Efficient Fine Tuning for Fashion Object Detection

**DOI:** 10.3390/s23136083

**Published:** 2023-07-01

**Authors:** Benjiang Ma, Wenjin Xu

**Affiliations:** School of Information Science and Technology, Qingdao University of Science and Technology, Qingdao 266061, China

**Keywords:** object detection, fine tuning, clothing dataset

## Abstract

Pre-trained models have achieved success in object detection. However, challenges remain due to dataset noise and lack of domain-specific data, resulting in weaker zero-shot capabilities in specialized fields such as fashion imaging. We addressed this by constructing a novel clothing object detection benchmark, Garment40K, which includes more than 140,000 human images with bounding boxes and over 40,000 clothing images. Each clothing item within this dataset is accompanied by its corresponding category and textual description. The dataset covers 2 major categories, pants and tops, which are further divided into 15 fine-grained subclasses, providing a rich and high-quality clothing resource. Leveraging this dataset, we propose an efficient fine-tuning method based on the Grounding DINO framework to tackle the issue of missed and false detections of clothing targets. This method incorporates additional similarity loss constraints and adapter modules, leading to a significantly enhanced model named Improved Grounding DINO. By fine-tuning only a small number of additional adapter module parameters, we considerably reduced computational costs while achieving performance comparable to full parameter fine tuning. This allows our model to be conveniently deployed on a variety of low-cost visual sensors. Our Improved Grounding DINO demonstrates considerable performance improvements in computer vision applications in the clothing domain.

## 1. Introduction

The fashion industry is a vast and continuously evolving domain where computer vision technology is becoming increasingly indispensable. Although tasks associated with fashion object detection may share similarities with those found in general computer vision benchmarks, the fashion industry’s unique intricacies and domain-specific terminology warrant a tailored approach. Object detection is paramount in various applications, such as clothing product search [1] and virtual try-on [2,3]. Consequently, it is crucial to develop a specialized object detector explicitly designed for the fashion domain, which can contribute to advancing the field and improving real-world applications.

**Figure 1:**
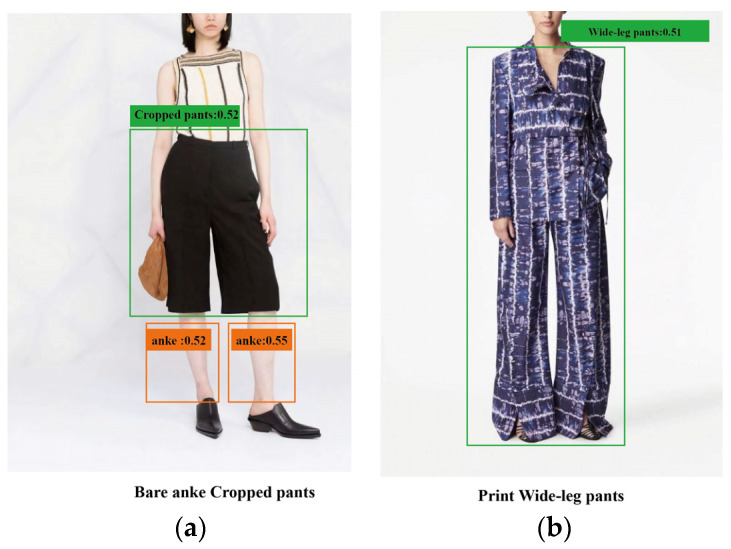
Illustrative examples of the performance limitations of the Grounding DINO model: (**a**) a case where the model is easily misled and identifies “ankles” and “cropped pants” without grasping the semantic essence; (**b**) an instance of the model’s confusion when dealing with identical styles of clothing, leading to the entire model being inaccurately encapsulated.

There are two primary methods for object detection: closed-set object detection, which can only detect known categories in a dataset, and open-set object detection, which can detect arbitrary objects specified by human language input. This paper addresses the challenges faced by these methods in the fashion image domain and introduces a new garment object detection benchmark, Garment40K. Closed-set object detection models such as Mask R-CNN [4] and DETR [5] are limited by their reliance on additional annotated data to generalize to new visual concepts and domains. In the fashion image domain, these models encounter difficulties due to fine-grained categories and the lack of extensive annotated data for training large-scale models. Conversely, open-set object detection algorithms, like ViLD [6], leverage visual and linguistic knowledge distillation techniques to align textual and image embeddings, surpassing supervised models of the time. Building upon these foundations, GLIP [7] unifies object detection and phrase grounding as pre-training tasks, learning object-level, language-aware, and semantically rich visual representations. Grounding DINO [8], a recently proposed model, inherits GLIP’s advantages, optimizing end-to-end detection without complex modules and demonstrating state-of-the-art object detection performance. However, these models still exhibit inaccuracies during inference, particularly in the fashion image domain, where they struggle with zero-shot detection and misidentification. The performance limitations of Grounding DINO are markedly illustrated with the examples depicted in Figure 1a,b. The model demonstrates a marked susceptibility to confusion when processing a fashion dataset, a phenomenon that is not uncommon and is worthy of closer examination. In the instance outlined in Figure 1a, Grounding DINO is easily misled by text, identifying “ankle” and “cropped pants” for “Bare ankle cropped pants” without grasping the semantic essence. The situation escalates in Figure 1b, where the model inaccurately encapsulates the entire model, evidencing its propensity for disarray when confronted with identical styles of apparel, such as tops and pants. The root of these issues can be traced back to the datasets used for pre-training these models. Inherently exhibiting a long-tail distribution, these datasets create a deficit in the model’s understanding of images related to the fashion domain. This shortcoming is further compounded by the inferior quality of many text descriptions in large-scale image–text datasets. Despite their extensive lexical range, these datasets enhance the model’s vulnerability to linguistic obfuscation, leading to further inaccuracies in image recognition and interpretation.

To address these limitations, we introduce Garment40K, a carefully designed clothing object detection benchmark. This dataset comprises clothing images, corresponding short textual descriptions, and images of models wearing garments from three angles, with major categories such as pants and tops. Garment40K is a comprehensive, professional, and high-quality fashion domain resource. The Garment40K dataset offers several distinct advantages over previous datasets:

- Comprehensiveness: The images in Garment40K are extensively annotated with categories, text descriptions, and bounding boxes. The dataset covers 15 fine-grained clothing categories and includes richly varied perspectives of human images. This level of comprehensive and detailed information is not commonly found in existing datasets, making Garment40K valuable for various computer vision tasks related to clothing analysis.
- Scale: Garment40K has the potential to be the largest standalone dataset specifically designed for clothing object detection. With 40,000 clothing images, it provides a significant number of diverse and labeled data for training and evaluating clothing detection models. This larger scale enables researchers to develop more robust and accurate algorithms for detecting and recognizing garments in images.
- Availability: Garment40K is intended to make the dataset publicly accessible to the research community. By making it widely available, we aim to foster advancements in clothing object detection research. This availability promotes collaboration, encourages innovation, and allows researchers from around the world to benefit from the dataset, leading to potential breakthroughs in the field.
- Potential: Garment40K has the potential to serve as a high-quality dataset for human image generation tasks. Additionally, as a future research direction, the dataset can be further enhanced by annotating human parsing labels and including human pose representations. This expansion would transform Garment40K into a multimodal clothing dataset, facilitating research in areas such as fashion recommendation systems, virtual try-on technologies, and other applications that require detailed clothing analysis in conjunction with human pose estimation.

Furthermore, we introduce similarity loss to augment the supervision signals, effectively leveraging real clothing images as image guidance. This approach enhances the accuracy of the model in detecting clothing objects by utilizing the informative cues present in clothing images. As model sizes continue to grow, fine tuning all model parameters becomes infeasible. Additionally, due to computational and storage costs, independently storing and deploying fine-tuned models becomes prohibitively expensive, making it difficult for small- and medium-sized enterprises in the clothing domain to bear the costs. Aiming to resolve these two issues, we fine-tuned pre-trained models using adapter tuning [9] on the Garment40K dataset, inspired by the works of parameter-efficient transfer learning methods. We incorporated adapter modules into Grounding DINO’s feature enhancer layer and decoder layer, which enables the model to effectively learn novel linguistic and visual information. This approach leads to an efficient learning process while minimizing both the computational burden and deployment costs, ultimately resulting in enhanced model performance within the fashion domain. Our contributions can be summarized as follows:

1. We introduce Garment40K, a novel open-set clothing object detection benchmark, providing a valuable resource for the fashion domain.
2. We employ parameter-efficient transfer learning methods for fine-tuning Grounding DINO, integrating adapter modules to enable the effective learning of new vocabulary.
3. We introduce an additional similarity loss function that serves as a supplementary supervision signal to enhance the model’s learning of the object detection task.
4. We address the limitations of state-of-the-art open-set object detection models in the fashion image domain by adapting Grounding DINO for improved performance.

## 2. Related Work

### 2.1. Clothing Datasets

Developing high-quality, large-scale annotated datasets has driven significant advancements in object detection in the fashion domain. DeepFashion [10] and VITON-HD [11] are two prominent examples of such datasets, each with strengths and weaknesses. DeepFashion is a large-scale clothing dataset consisting of over 800,000 comprehensively annotated images. Each image features 50 categories and 1000 descriptive attributes, organized into 5 groups: texture, fabric, shape, part, and style. Although this dataset has facilitated the development of various successful object detectors, its reliance on manually cleaned web-collected data and annotators without specialized fashion expertise raises concerns about data quality. On the other hand, VITON-HD is a 1024 × 768 virtual try-on dataset comprising 13,679 pairs of high-quality, front-facing images of top garments. While the data are presented uniformly, the dataset is limited by its small size and lack of image descriptions. The DeepFashion-MultiModal [12] dataset has recently emerged, offering a high-quality, large-scale human body dataset with rich multimodal annotations for text-driven human image generation and text-guided human image processing. However, it does not explicitly address open-set clothing object detection tasks. In response to these challenges, we propose the Garment40K dataset, combining the advantages of DeepFashion and VITON-HD while minimizing their drawbacks. This dataset offers a more comprehensive and suitable solution for open-set clothing object detection, ensuring better coherence and utility for research and development purposes.

### 2.2. Open-Set Object Detection

Recent advancements in open-set object detection have been driven by works such as CLIP [13] and ALIGN [14], which focus on large-scale collection of image–text pairs and joint image–text model training using contrastive learning. Although these works excel in image-level open-vocabulary recognition, our study emphasizes detecting objects with arbitrary text input. A variety of approaches have been developed to address the challenges of open-set object detection, which leverages linguistic generalization to detect any arbitrary class. For instance, OV-DETR [14] uses image and text embeddings encoded by the CLIP model as queries within the DETR [5] framework to decode class-specific boxes. ViLD [6], on the other hand, extracts knowledge from the CLIP teacher model for R-CNN class detectors [15,16,17], ensuring the incorporation of linguistic semantics in learned region embeddings. Another notable method, GLIP [7] redefines object detection as a grounding problem and employs additional grounding data to facilitate the learning of aligned semantics at phrase and region levels. This approach outperforms fully supervised detection benchmarks. Similarly, DetCLIP [6,18] utilizes large-scale image–caption datasets and generated pseudo-labels to expand its knowledge base, leading to improved detector generalization. Lastly, Grounding DINO [8] enhances open-set object detection by implementing visual-language modality fusion across multiple stages. This deep fusion strategy effectively boosts the detection ability for open-set targets. In summary, these diverse methods illustrate the range of techniques available for tackling open-set object detection challenges while maintaining the standards of academic writing. While these studies predominantly concentrate on general open-vocabulary detection, it is crucial to customize general models for distinct scenarios by incorporating specialized data and guidelines. Our research endeavors to refine these general models explicitly for the fashion domain, with the objective of augmenting detection quality and ensuring practical, controlled inputs and outputs aligned with professional requirements.

### 2.3. Parameter-Efficient Transfer Learning

Transfer learning from pre-trained models is a popular paradigm in computer vision, yielding impressive performance across various tasks. However, fine tuning separate models for each task can be computationally expensive, particularly for large-scale models. To alleviate this concern, several studies [19,20,21,22] have introduced lightweight alternatives that involve updating a minimal number of additional parameters while maintaining the majority of pre-trained parameters in a frozen state. Houlsby et al. proposed adapter tuning [6], a method that incorporates small neural modules, referred to as adapters, into each layer of the pre-trained network. During the fine-tuning process, only these adapters undergo training. At present, the use of adapter modules is virtually non-existent in downstream tasks like object detection. We incorporated adapter modules into our object detection method at appropriate positions, thereby enhancing performance and reducing the number of adjustable parameters.

## 3. The Garment40K Dataset

**Figure 2:**
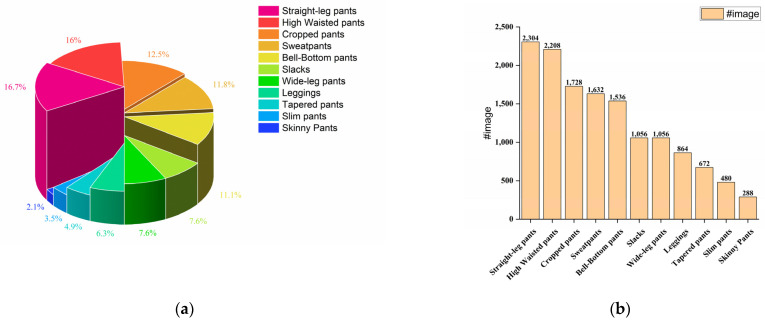
Comparative analysis of different types of pants in the dataset. (**a**) The bar chart showcases the absolute quantity of each type of pants in the dataset; (**b**) the pie chart displays the relative percentage distribution of the various types of pants.

**Figure 3:**
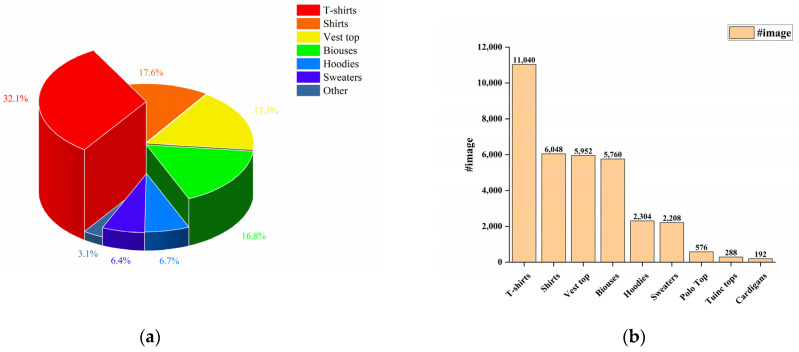
Comparative analysis of different types of tops in the dataset. (**a**) The bar chart represents the absolute quantity of each type of top in the dataset; (**b**) the pie chart illustrates the relative percentage distribution of the different types of tops.

**Figure 4:**
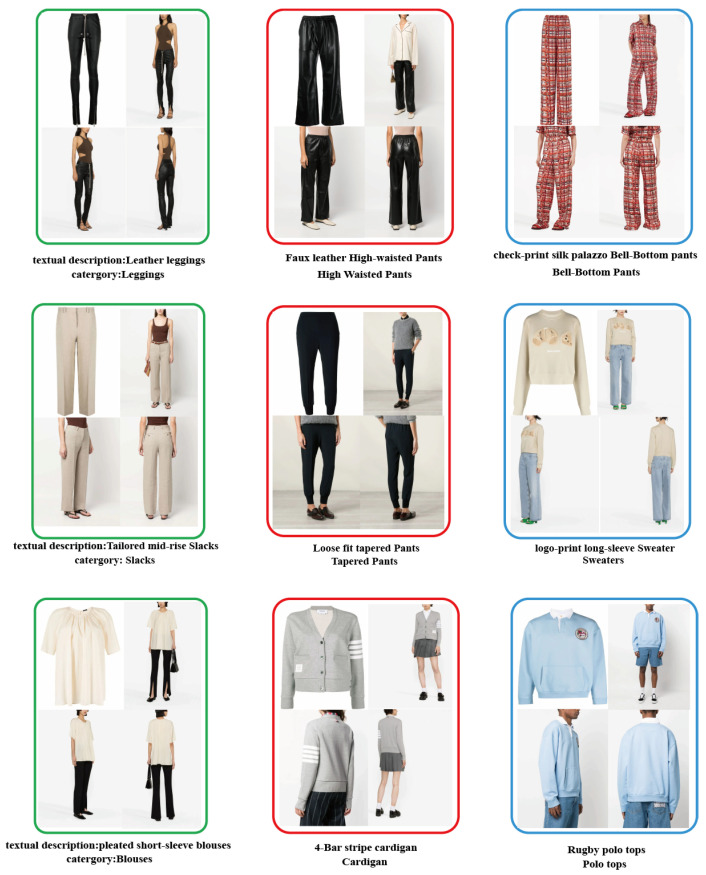
Samples of specific image collections from the Garment40K dataset.

We provide the community with the Garment40K dataset, which possesses several enticing characteristics. Firstly, it boasts exceptional image quality and uniform dimensions and encompasses over 140,000 fashion images. Secondly, it serves as benchmark for many tasks, such as object detection, clothing classification, and human image generation, among others. This dataset comprises 2 subclasses: pants and tops, with pants encompassing 11 major categories and tops containing 9. Figure 2 and Figure 3 provide a detailed distribution of the quantities of various types of tops and pants in the dataset. We define a set of four images as a single collection, featuring garment and three distinct perspectives of models adorning the attire (front, rear, and side). As demonstrated in Figure 4, some specific instances of these collections are presented to provide a clear understanding of our data integration approach for multi-angle clothing. The dataset encompasses over 40,000 such collections in total. Moreover, each garment corresponds to a textual description encompassing attributes such as texture, fabric, shape, and style. Furthermore, all frontal images of garments were detected, including a JSON file containing bounding box labels and box locations, which serve as a supervised benchmark for object detection.

### Image Collection

E-commerce websites are prevalent sources for creating clothing datasets. Apart from this source, we enriched and expanded the image collection with our own garment photographs. Approximately 70% of images were scraped from shopping websites, while the remaining 30% were manually captured. For images gathered from the internet, human annotators were instructed to discard unusable images with low resolution or poor quality, or those where the primary subject was unrelated to clothing. Additionally, images that failed to meet the requirements of a single collection were removed. All images were uniformly cropped to 480 × 640 pixels, ensuring that both human subjects and garments were unobscured. In total, over 140,000 images were retained, comprising 40,000 collections. We also cleaned the textual descriptions by removing brand names and irrelevant symbols to prevent copyright infringements. Upon completing the image collection process, we employed Grounding DINO [5] to conduct preliminary detection on our images, performing inference without updating the model’s weights. A significant number of bounding boxes were present in the inference results, which were manually rectified.

## 4. Method

**Figure 5:**
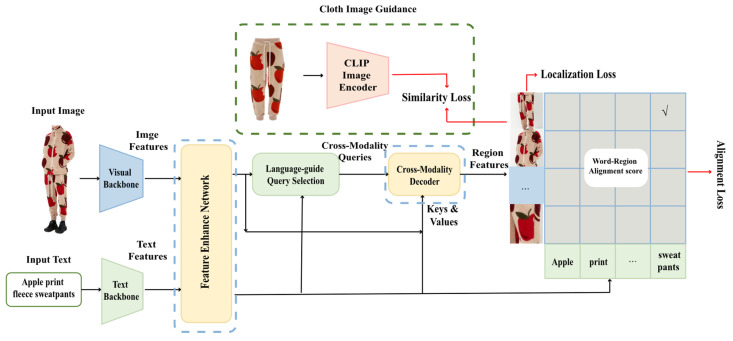
The framework of Improved Grounding DINO. In the figure, the module within the blue dotted box is the feature enhancer network and cross-modality decoder, which were improved based on the original architecture of Grounding DINO. The feature enhancer network is composed of the enhancer–adapter layer proposed in this paper, while the cross-modality decoder is built with the decoder–adapter layer newly introduced in this paper. In addition, the module within the green dotted box represents the clothing image guidance newly introduced in this paper. Apart from that, the other parts of the model are consistent with the original architecture of Grounding DINO.

In order to ascertain the validity of the Garment40K dataset while concurrently diminishing the necessity for extensive model fine tuning, we meticulously adapted the Grounding DINO [8] model. This led to the inception of a simpler version of the architecture, hereby designated as “Improved Grounding DINO”. Within this section, a concise overview of Grounding DINO is initially presented in Section 4.1. This is followed by an explication on the integration of adapters within the feature enhancer layer of Grounding DINO, giving rise to a novel layer, designated as feature enhancer–adapter layer, as elaborated in Section 4.2. Correspondingly, Section 4.3 sheds light on the integration of adapters within the decoder layer of Grounding DINO, leading to the inception of what is termed decoder–adapter layer. Finally, Section 4.4 details the integration of auxiliary clothing image guidance. This component, which is principally based on similarity loss, fortifies the architecture by providing additional supervision signals. The comprehensive framework can be seen in Figure 5.

### 4.1. Grounding DINO

Grounding DINO [8], a state-of-the-art object detection model, possesses pre-trained models that have undergone extensive training on large-scale datasets, exhibiting robust transferability to downstream tasks. Consequently, it is posited that these models can effectively model clothing object detection with minimal fine tuning. The overall workflow and structure largely remain the same as depicted in Figure 5 but without incorporating the clothing image guidance represented by the green dotted line. Grounding DINO first extracts rudimentary image and text features using an image backbone and a text backbone, respectively. These rudimentary features are then input into the feature enhancer network for cross-modality feature fusion. Upon acquiring cross-modality text and image features, a language-guided query selection module is employed to select cross-modality queries from the image features. Similar to object queries in most DETR-like models [5,23,24], these cross-modality queries are fed into a cross-modality decoder, which probes desired features from the bimodal features and updates itself accordingly. Finally, the output queries of the decoder layer are utilized to predict object boxes and extract corresponding phrases.

### 4.2. Feature Enhancer–Adapter Layer

**Figure 6:**
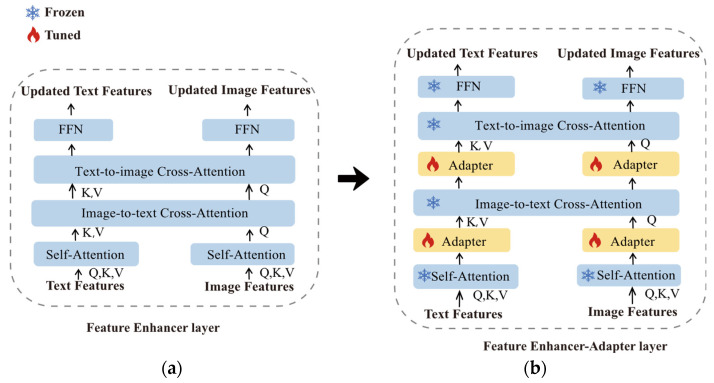
Visualization of the improvement process. (**a**) Feature enhancer layer in Grounding DINO. (**b**) Feature enhancer–adapter layer in Improved Grounding DINO.

The adapter [9] features a bottleneck architecture composed of two fully connected (FC) layers and an intermediate GELU activation layer. The first FC layer projects the input to a lower dimension, while the second FC layer reverts it back to the original dimension. Within the Grounding DINO architecture, a critical constituent is the feature enhancer network, which originally solely consists of the stacked feature enhancer layer, as depicted in Figure 6a. In order to perform effective fine tuning, we made improvements to the feature enhancer layer: First, we introduced an adapter following both the text self-attention layer [25] and the image self-attention layer [26]; subsequently, we integrated two parallel adapters for image-to-text cross-attention. As illustrated in Figure 6b, we term this feature enhancer–adapter layer. The computation for the feature enhancer–adapter layer can be formulated as follows:

(1) $$O_{l}^{T_{1}}=\mathrm{Adapter}\left(\mathrm{MSA}\left(I_{l-1}\right)\right),$$

(2) $$O_{l}^{I_{1}}=\mathrm{Adapter}\left(\mathrm{MSA}\left(T_{l-1}\right)\right),$$

(3) $$K_{T},V_{T},Q_{I}=W_{l}^{K}O_{l}^{T_{1}},W_{l}^{V}O_{l}^{T_{1}},W_{l}^{Q}O_{l}^{I_{1}},$$

(4) $$O_{l}^{T_{2}}=\mathrm{Adapter}\left(\mathrm{ITCA}\left(Q_{I},K_{T},V_{T}\right)\right),$$

(5) $$O_{l}^{I_{2}}=\mathrm{Adapter}\left(\mathrm{ITCA}\left(Q_{I},K_{T},V_{T}\right)\right),$$

(6) $$T_{l}=\mathrm{FNN}\left(\mathrm{TICA}\left(O_{l}^{T_{2}}\right)\right),$$

(7) $$I_{l}=\mathrm{FNN}\left(\mathrm{TICA}\left(O_{l}^{I_{2}}\right)\right).\;$$

In the formula mentioned above, several acronyms are used to represent different components. MSA stands for multi-head self-attention mechanism, which is a type of attention mechanism. Additionally, ITCA and TICA are used to represent image-to-text cross-attention and text-to-image cross-attention, respectively. Lastly, FFN is an abbreviation for feed-forward neural network.

In the (l−1)-th layer of the feature enhancer–adapter layer, the text and image output features, represented as $O_{l}^{T_{1}}$ and $O_{l}^{I_{1}}$, are passed through their corresponding initial adapter modules and MSA to generate hidden vectors $\;O_{l}^{I_{1}}$ and $O_{l}^{T_{1}}$. The text output features are fed to a linear projection layer, producing vectors $K_{T}$ and $V_{T}$, while the image output features undergo MLP to generate vector $Q_{I}$. All features are fed to ITCA and then separately enter their respective secondary adapter modules, resulting in the hidden $O_{l}^{T_{2}}$ and $O_{l}^{I_{2}}$. Finally, the features are processed with TICA and FNN [25], obtaining inputs $I_{l}$ and $T_{l}\;$for the l-th layer.

### 4.3. Decoder–Adapter Layer

**Figure 7:**
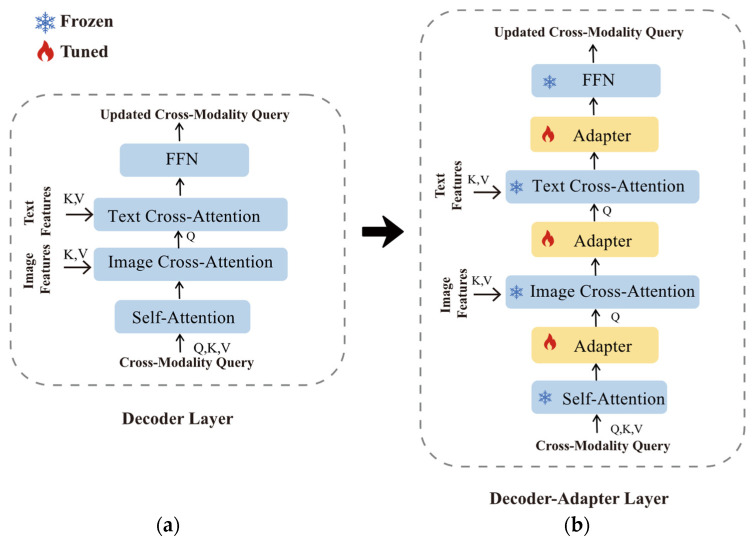
Visualization of the improvement process. (**a**) Decoder layer in Grounding DINO. (**b**) Decoder–adapter layer in Improved Grounding DINO.

Within the Grounding DINO architecture, another critical component is the cross-modal decoder, which originally only consists of the decoder layers depicted in Figure 7a. We made the following improvements to it: We successively integrated three adapter modules, and these were sequentially placed after multi-head self-attention, image cross-attention, and text cross-attention, respectively. As illustrated in Figure 7b, we refer to this arrangement as decoder–adapter layer. The computation for the decoder–adapter layer can be expressed as follows:

(8) $$O_{l}^{1}=\mathrm{Adapter}\left(\mathrm{MSA}\left(I_{l-1}\right)\right),$$

(9) $$Q_{l}^{1}=W_{l}^{Q}O_{l}^{1},$$

(10) $$O_{l}^{2}=\mathrm{Adapter}\left(\mathrm{ICA}\left(Q_{l}^{1},K_{I},V_{I}\right)\right),$$

(11) $$Q_{l}^{2}=W_{l}^{Q}O_{l}^{2},$$

(12) $$O_{l}^{3}=\mathrm{Adapter}\left(\mathrm{TCA}\left(Q_{l}^{2},K_{T},V_{T}\right)\right),$$

(13) $$I_{l}=\mathrm{FFN}\left(O_{l}^{3}\right).$$

In the formula mentioned above, ICA and TCA are used to represent image cross-attention and text cross-attention. $O_{l}^{1}$, $O_{l}^{2}$, and $O_{l}^{3}$ are the hidden outputs after passing through the adapter module, and the final output of the FFN is input $I_{l}$ for the next layer. During training, all the other layers of the transformer model are frozen while only the adapters are updated.

### 4.4. Loss Function

Expanding upon the foundations established by previous DETR-like frameworks, our method adopts L1 loss and GIOU loss [27] for bounding box regressions, which constitute the localization loss. Moreover, we incorporated GLIP [7], implementing the alignment loss between predicted objects and language tokens for classification. Alignment loss is essentially contrastive learning loss. To calculate the final loss of the model, it is necessary to match the model’s predicted results and the ground truth. This matching problem can be viewed as a bipartite graph matching problem, which can be effectively solved using the Hungarian algorithm. Before applying the Hungarian algorithm, the key is to build a cost matrix to quantify the bipartite graph matching loss between the model predictions and the ground truth. Each element in the cost matrix is obtained by considering the appropriate weighted localization loss and alignment loss. Once the Hungarian algorithm is used to find the matching between model predictions and ground truth that minimizes the total bipartite matching loss, localization loss and alignment loss are calculated based on the matched predictions and ground truth. In addition, we creatively added additional similarity loss to provide supplementary supervision signals to help the model better learn the object detection task. The Garment40K dataset includes real clothing images, which can serve as clothing image guidance. As shown in the green dotted line in Figure 5, the clothing image is extracted using a frozen CLIP image encoder to generate a clothing feature. The CLIP image encoder has a strong ability to extract meaningful and informative features from images by training on a large-scale dataset of image–text pairs. This feature extraction process is offline, and all the clothing image features are extracted before training and only used directly during the training process, not working during inference. Each predicted bounding box corresponds to a “region feature” (the final layer feature map of the cross-modality decoder), and the region feature corresponding to the predicted bounding box obtained with optimal matching is compared with the clothing feature using similarity loss calculation. This similarity loss helps ensure that the features extracted for the detected objects closely align with those of the real clothing images. Ultimately, the loss function is defined as a linear combination of alignment loss ($L_{\mathrm{align}\;}$), localization loss ($L_{\mathrm{loc}}$), and similarity loss ($L_{\mathrm{sim}}$), denoted by L:

(14) $$L=L_{\mathrm{sim}}+L_{\mathrm{loc}}+L_{\mathrm{align}}.$$

We use p to represent the feature vector of the clothing object, and$\;\hat{p}={\left\{{\hat{p}}_{i}\right\}}_{i=1}^{N}$ as the region feature corresponding to the optimal match. It is assumed that N is the number of objects in the image. The overall loss, $\begin{array}{c} L_{sim} \end{array}$, can be expressed as $\begin{array}{c} \overset{N}{\underset{i}{\sum }}L_{sim}\left(p,{\hat{p}}_{i}\right) \end{array}$. The calculation formula for the similarity loss function $\begin{array}{c} L_{sim}\left(p,{\hat{p}}_{i}\right) \end{array}$ is as follows:

(15) $$\begin{array}{c} \begin{array}{c} L_{sim}\left(p,{\hat{p}}_{i}\right) \end{array}=1-max{\left(0,\frac{p\cdot {\hat{p}}_{i}}{∥p∥∥{\hat{p}}_{i}∥}\right)}^{k}\; \end{array}$$

where $\frac{p\cdot {\hat{p}}_{i}}{∥p∥∥{\hat{p}}_{i}∥}\;$represents the cosine similarity between p and ${\hat{p}}_{i}$; by computing the maximum value between 0 and this term, the non-negativity of the resulting value is ensured. Cosine similarity provides a means to capture the angular relationship between feature vectors in a normalized and robust way, particularly in high-dimensional spaces. This makes it well-suited for comparing semantic similarities among different representations. K serves as a hyperparameter, facilitating the adjustment of the similarity magnitude between two vectors, thereby effectively quantifying the disparity between them. For example, when k is set to 1 and the cosine similarity between vectors p and $\hat{p}\;$ is set to 1/2, with similarity loss of 1/2, this result does not adequately represent the considerable difference between them. The cosine similarity result is too large, resulting in excessive tolerance. By increasing the k value, this issue can be addressed, allowing the similarity loss to more precisely depict the difference between the two vectors. In our study, we chose a value of 60 for hyperparameter k.

## 5. Experiments

### 5.1. Setup

The Garment40K dataset is systematically divided into three distinct subsets, with 80% allocated for training, 10% designated for validation, and the remaining 10% reserved for testing. Careful consideration is given to prevent any overlap between the fine-tuning and test sets. Our Improved Grounding DINO model commences with the incorporation of pre-trained weights from the Grounding DINO [8] model, followed by architectural refinements and subsequent fine tuning using the Garment40K dataset. Key configuration parameters, including warm-up epochs, batch size, learning rate, and weight decay, were set to 2, 64, 9 × 10^−3^, and 0.02, respectively. The training of the Improved Grounding DINO model was conducted across two Nvidia A100 GPUs, spanning a total of five epochs.

### 5.2. Metric

In the experimental evaluation, we adopted average precision (AP) as the cardinal metric for the assessment of the performance exhibited by the object detection models. AP elegantly amalgamates the virtues of precision and recall into a solitary score, thereby providing a holistic quantification of the model’s effectiveness:

1. Precision: Precision is rigorously defined as the ratio of the cardinality of true positive predictions to the sum total of instances flagged as positive by the model. It is a critical measure of the veracity of the model in terms of positive identification.
  (16) $$\mathrm{Precision}=\frac{\mathrm{True}\;\mathrm{Positives}}{\mathrm{True}\;\mathrm{Positives}+\mathrm{False}\;\mathrm{Positives}}$$
2. Recall: On the contrary, recall is the ratio of the cardinality of true positive predictions to the entirety of instances that are genuinely positive. It offers insights into the model’s capability of correctly identifying positive instances.
  (17) $$\mathrm{Recall}=\frac{\mathrm{True}\;\mathrm{Positives}}{\mathrm{True}\;\mathrm{Positives}+\mathrm{False}\;\mathrm{Negatives}}$$
3. Precision–recall curve: Pertaining to object detection, it is customary to have detections accompanied by a confidence score. By judiciously adjusting the threshold of this confidence score, a gamut of precision and recall values is obtained. The precision–recall (PR) curve is manifested by plotting these values over an array of thresholds.
4. Average precision (AP): AP is a quintessential representation of the morphology of the precision–recall curve. It is deduced as the weighted mean of the precision values at each threshold, with the increment in recall from the antecedent threshold serving as the weight:

(18) $$\mathrm{AP}=\underset{n}{\sum }\left(R_{n}-R_{n-1}\right)\cdot P_{n},$$

where $P_{n}$ and $R_{n}$ represent precision and recall, respectively, at the *n*-th threshold.

### 5.3. Supervised Transfer on Garment40K

We conducted a comparative study on Improved Grounding DINO, GLIP [7], and Grounding DINO [8]. Our primary objective was to evaluate the transfer capabilities of these models on clothing targets using Garment40K. The Grounding DINO-T [8] model architecture, which incorporates Swin-T [28] as the image backbone, served as the foundation for developing the Improved Grounding DINO model. The model was fine-tuned on the Garment40k-Train dataset, as were the other baseline models. Table 1 provides quantitative comparisons with the AP metric. Throughout the training process, only the parameters of the adapter module were updated, leaving the remaining parameters frozen. Our Improved Grounding DINO, updating merely 1.3 M parameters, achieved 43.5 in AP on Garment40K-Test and 43.2 in AP on Garment40K-Val. Remarkably, it surpassed the current state-of-the-art, open-set, fully parameter-fine-tuned Grounding DINO and the GLIP model on Garment40K. The occurrence of this result can be attributed to the fact that when large models such as GLIP and Grounding are fully fine-tuned using a small number of data, it leads to catastrophic forgetting. Interestingly, models that do undergo the fine tuning of a small number of parameters tend to perform better. As the scale of pre-trained data increased, the performance of our model improved. We believe that by combining larger Grounding DINO [8] models, increasing the number of training data, and scaling up learnable parameters, Improved Grounding DINO could be further enhanced.

**Figure 8:**
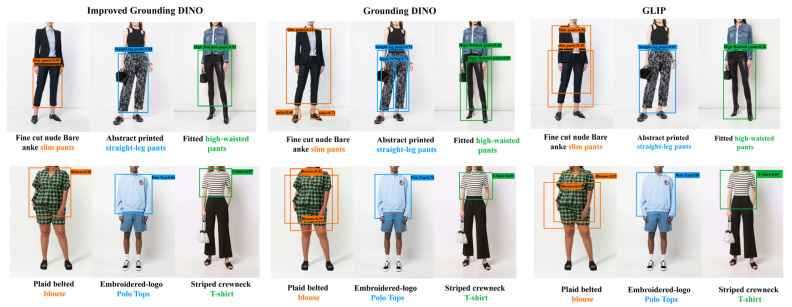
Comparison with Grounding DINO and GLIP on Garment40K-Test. Phrases and corresponding boxes are matched with the same colors.

We also provide visual qualitative comparisons with all baselines on Garment40K-Test in Figure 8. We observed a marked distinction in the performance of Grounding DINO and GLIP on Garment40K. Specifically, the efficacy of both models was remarkably high in instances such as “Striped crewneck T-shirt” and “Embroidered Polo Tops”. Nonetheless, there were instances where images were prone to perturbations emanating from the text, as exemplified by “Fitted high-waisted pants” and “Abstract printed straight-leg pants”. Our model is better able to understand the semantic information of the text, capture the main entities of the text, and avoid false detections. In addition, our model can reduce the occurrence of large bounding boxes, thus achieving more precise bounding boxes. Of course, there is still room for improvement.

### 5.4. Zero-Shot Transfer on COCO and LVIS

We fine-tuned our model on the Garment40K dataset to evaluate the impact on the transferability of our model to both common and rare object categories in the COCO and LVIS datasets. COCO [28] object detection encompasses 80 common object classes. As presented in Table 2, the performance of Improved Grounding DINO on the 2017-val set was virtually equivalent to that of Grounding DINO-T [8]. Under the least favorable conditions, the Improved Grounding DINO model exhibited a performance decrement of 0.3 AP compared with the Grounding DINO model. Nevertheless, in the majority of cases, the Improved Grounding DINO model outperformed the Grounding DINO model, achieving an increase of 0.1 to 0.2 in AP. We think that the transfer capabilities for widespread categories remain unaffected after fine tuning. LVIS [29], a dataset specifically curated for long-tail objects, encompasses over 1000 categories for evaluation. The experimental findings, as presented in Table 3, reveal varying degrees of transferability degradation to rare object categories after fine tuning, with a maximum decline of 1.1 in AP. These results underscore the considerable impact of fine tuning on the detection of rare categories, thereby highlighting it as a promising avenue for further research exploration.

### 5.5. Ablations

In this section, we present an ablation study that we conducted to analyze the impact of specific components on the model’s performance by progressively removing or altering them in the experimental setup. We focused on three aspects: First, we employed the adapters within the feature enhancer and cross-modal decoder individually to investigate their roles in the Improved Grounding DINO model. Second, we removed the auxiliary similarity loss function to evaluate its contribution to the model’s performance. All models underwent fine tuning utilizing the Garment40k dataset. For those employing the adapter module, parameter updating was exclusively confined to the adapter module, while the remainder of the parameters were rendered static. The results are presented in Table 4. We observed that without similarity loss, the performance on Garment40K-Test was 1.1 lower in AP than that of Improved Grounding DINO. When using feature enhancer–adapter and decoder–adapter individually, the performance was 0.3 to 0.7 lower in AP than with similarity loss. Eliminating the adapters in the feature enhancer and cross-modal decoder led to a decline of 1.4 to 1.6 in AP in the results.

**Figure 9:**
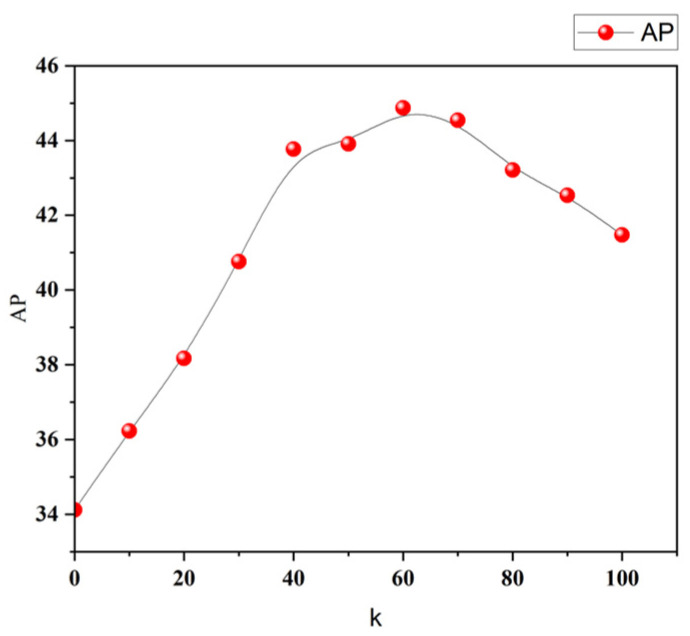
Relationship between hyperparameter k and AP. The graph illustrates the influence of different k values on the model’s accuracy. An optimal k value, approximately around 50, is observed, beyond which the model’s accuracy begins to deteriorate.

We evaluated the impact of different k values (hyperparameters in the cosine similarity loss) on the performance of the model. This aids in identifying the optimal k value to more accurately quantify the differences between feature vectors during the training process. As illustrated in Figure 9, interestingly, we discovered that there exists an optimal k value around 60, beyond which the model’s accuracy declines.

## 6. Conclusions

In this study, we introduce a novel and challenging benchmark for clothing open object detection, named Garment40K, which serves as an effective metric to assess various object detection techniques. Leveraging the Garment40K training set, we present the Improved Grounding DINO model, incorporating adapter components and additional loss functions. This approach significantly reduces training costs compared with fully fine-tuned object detection models while achieving competitive or superior performance. Our model attained an impressive 43.5 score in AP on the Garment40K test set. Despite the many benefits, a limitation of our approach lies in its potential inadequacy in addressing particularly complex object detection scenarios due to its simplified strategy. In future work, we may adopt a parameter-efficient fine-tuning method on larger-scale pre-trained models to enhance the model’s robustness.

## Figures and Tables

**Table 1:** Supervised transfer on Garment40K.

**Model**	**Pre-Training Data**	**Fine-Tuning Data**	**Tunable** **Param (M)**	**Inference**	
				**Garment40K-Test**	**Garment40K-Val**
GLIP-T	O365, GoldG, Cap4M	Garment40K	232	42.5	42.8
	O365, GoldG		232	41.9	42.3
Grounding DINO-T	O365	Garment40K	172	41.5	41.6
	O365, GoldG		172	42.4	42.7
	O365, GoldG, Cap4M,		172	42.8	42.6
Our model	O365	Garment40K	1.3	41.7	41.8
	O365, GoldG		1.3	42.8	42.9
	O365, GoldG, Cap4M		1.3	43.2	43.5

**Table 2:** Zero-shot domain transfer on COCO.

Model	Pre-Training Data	Fine-Tuning Data	Zero-Shot 2017val
Our model	O365	Garment40K	46.4
	O365, GoldG		47.9
	O365, GoldG, Gap4M		48.5
Grounding DINO-T	O365	None	46.7
	O365, GoldG		48.1
	O365, GoldG, Cap4M		48.4

**Table 3:** Zero-shot domain transfer on LVIS.

Model	Pre-Training Data	Fine-Tuning Data	MiniVal			
			APr	APc	APf	AP
Our model	O365, GoldG	Garment40K	12.9	18.3	31.3	24.6
	O365, GoldG, Gap4M		46.5	17.5	22.8	31.6
Grounding DINO-T	O365, GoldG	None	14.4	19.6	32.2	25.6
	O365, GoldG, Cap4M		48.4	18.1	23.3	32.7

**Table 4:** Ablations for our model.

Model	Inference Garment40k-Test
Feature enhancer–adapter layer	41.4
Decoder–adapter layer	41.6
$\mathrm{Feature}\;\mathrm{enhancer}–\mathrm{adapter}\;\mathrm{layer}+L_{sim}$	42.1
$\mathrm{Decoder}–\mathrm{adapter}\;\mathrm{layer}+L_{sim}$	41.9
Feature enhancer–adapter + decoder–adapter layer	42.3
Improved Grounding DINO (our model)	43.5

## Data Availability

The datasets generated and analyzed in the current study are available from the GitHub repository at https://github.com/Ma-benjiang/improved-Grounding-DINO (accessed on 26 June 2023).
